# Ellagic Acid Alleviates Hepatic Oxidative Stress and Insulin Resistance in Diabetic Female Rats

**DOI:** 10.3390/nu10050531

**Published:** 2018-04-25

**Authors:** Simran Alexandria Polce, Cameron Burke, Lucas Martins França, Benjamin Kramer, Antonio Marcus de Andrade Paes, Maria Alicia Carrillo-Sepulveda

**Affiliations:** 1 Department of Life Sciences, College of Arts and Sciences, New York Institute of Technology, Old Westbury, NY 11568, USA; spolce@nyit.edu; 2 Department of Biomedical Sciences, New York Institute of Technology College of Osteopathic Medicine, Old Westbury, NY 11568, USA; cburke08@nyit.edu (C.B.); bkramer@nyit.edu (B.K.); 3 Laboratory of Experimental Physiology, Department of Physiological Sciences, Federal University of Maranhao, Sao Luis, MA 65080-805, Brazil; lucas.mf@ufma.br (L.M.F.); antonio.marcus@ufma.br (A.M.d.A.P.)

**Keywords:** Ellagic Acid, Goto-kakizaki (GK) rats, hepatic steatosis, insulin resistance, oxidative stress, HIF-α, p47-phox, NRF2, Type II Diabetes Mellitus (T2DM), antioxidant

## Abstract

Non-alcoholic fatty liver disease (NAFLD) affects more than 70% of patients with type 2 diabetes mellitus (T2DM) and has become one of the most common metabolic liver diseases worldwide. To date, treatments specifically targeting NAFLD do not exist. Oxidative stress and insulin resistance have been implicated in the pathogenesis of NAFLD in diabetes. Accordingly, the goal of this present study was to determine whether Ellagic acid (EA), a natural antioxidant polyphenol found in berries and nuts, mitigates hepatic oxidative stress and insulin resistance in T2DM rats, and thus alleviates NAFLD. Using adult female Goto Kakizaki (GK) rats, a non-obese and spontaneous model of T2DM, we found that EA treatment significantly lowered fasting blood glucose and reduced insulin resistance, as shown by a 21.8% reduction in the homeostasis model assessment index of insulin resistance (HOMA-IR), while triglyceride and total cholesterol levels remained unchanged. Increased hepatic lipid accumulation and oxidative stress present in diabetic GK rats was markedly reduced with EA treatment. This effect was associated with a downregulation of the NADPH oxidase subunit, p47-phox, and overexpression of NF-E2-related factor-2 (NRF2). Moreover, EA was able to decrease the hepatic expression of hypoxia-inducible factor (HIF-α), a transcription factor linked to hypoxia and hepatic steatosis. We further showed that EA treatment activated an insulin signaling pathway in the liver, as evidenced by increased levels of phosphorylated Akt (Ser 473). In conclusion, our results demonstrate that EA diminishes blood glucose levels and potently suppress NAFLD in diabetic rats via mechanisms that involve reductions in p47-phox and HIF-α, upregulation of NRF2 and enhancement of the Akt signaling pathway in the liver. Together, these results reveal that EA improves hepatic insulin sensitivity and lipid metabolism as a result of its antioxidant effects. This implies an anti-diabetic effect of EA with beneficial effects for the treatment of hepatic complications in T2DM.

## 1. Introduction

Type 2 diabetes mellitus (T2DM) is a chronic metabolic disease, classically characterized by hyperglycemia and insulin resistance. Studies have recently identified that around 70% of patients with T2DM present with non-alcoholic fatty liver disease (NAFLD), a complication of T2DM that remains without specific treatment [1,2]. Alarmingly, a significant number of patients with NAFLD have an increased risk of developing T2DM [3]. A tight association between NAFLD and long-term diabetic complications has increasingly been recognized. Evidence has shown that T2DM patients with NAFLD have a two-fold increased risk of developing vascular complications [4], a major, challenging obstacle in treating diabetic patients. Accordingly, it is speculated that treatment focused on tackling NAFLD in diabetic patients may also mitigate diabetic vascular dysfunction. To date, there have been no drug therapies approved specifically for NAFLD [5]. NAFLD is associated with hepatic steatosis or accumulation of fat, predominantly triglycerides, within hepatocytes, not caused by alcohol consumption. This prompts a pro-inflammatory cellular response in which NAFLD can advance to hepatic fibrosis and ultimately, liver failure. Despite the pathophysiological mechanisms underlying NAFLD not being well elucidated, studies have suggested that oxidative stress is one of the main factors contributing to hepatic insulin resistance and the subsequent development of NAFLD. Overproduction of reactive oxygen species (ROS) along with reduced antioxidant enzymatic defenses negatively interferes with insulin signaling, causing insulin resistance and increased triglyceride storage in the liver [6]. However, recent evidence has led to the proposal that endoplasmic reticulum stress also plays a role in the onset of NAFLD [7,8]. Considering the role of the endoplasmic reticulum in lipid synthesis, disturbances in this organelle also contribute to impaired lipid metabolism in the liver [9], which ultimately results in the exacerbation of insulin resistance. Nevertheless, hepatic accumulation of saturated fatty acids, such as diacylglycerol and ceramides, has indeed been proposed as a trigger of insulin resistance in the liver through oxidative and endoplasmic reticulum stress-based mechanisms [10,11,12]. It has been reported that oxidative stress contributes to hypoxia, which also plays a significant role in the development and progression of NAFLD [13]. This is supported by studies showing that oxidative stress activates HIF-α, a transcription factor implicated in hypoxia and hepatic lipid accumulation [14,15].

Clinical studies have attempted to use synthetic antioxidant therapies which have the potential to treat diabetic complications. However, these clinical trials have been neither effective, nor conclusive [16,17]. In recent years, natural antioxidants have gained increasing attention over synthetic ones because of their potential benefits and facilitated access through natural diets and dietary supplements. Ellagic acid (2,3,7,8-tetrahydroxy-chromeno [5,4,3-cde] chromene-5, 10-dione) (EA) is a natural polyphenolic compound that is commonly found in fruits, such as raspberries, pomegranates, grapes and blackcurrants as well as in nuts. Our group has recently shown that EA improves vascular function in blood vessels exposed to hyperglycemic conditions through the reduction of oxidative stress [18]. In rodent models of hepatic ischemia reperfusion and aging, EA has been demonstrated to have beneficial effects on the liver by decreasing oxidative stress [19,20]. New insights into the therapeutic effects of EA in diabetic liver have been recently reported [21]; however, the mechanisms of these effects remain unclear.

Thus, this present study aims to examine whether EA reduces hepatic oxidative stress and insulin resistance in Goto Kakizaki rats, a non-obese model of T2DM widely utilized to study diabetic complications, and consequently, ameliorates NAFLD and possibly, vascular dysfunction.

## 2. Materials and Methods

### 2.1. Reagents

Ellagic acid (E2250), Dihydroethidium (DHE) methyl-beta-cyclodextrin (332615), acetylcholine (A6625) and D-glucose (G7021) were obtained from Sigma-Aldrich (St. Louis, MO, USA). The optimum cutting temperature compound (OCT), Tissue-Tek, was obtained from Sakura Finetek (Torrance, CA, USA).

### 2.2. Animals and Experimental Protocols

Goto-Kakizaki (GK) female rats, aged 11 months, were obtained from Taconic Farms (Germantown, NY, USA). The rats were housed at 25 ± 1 °C and maintained on a 12 h light–dark cycle, with free access to drinking water and a standard chow diet (Zeigler Rodent Chow NIH-31M) *ad libitum*. The GK rats were randomly allocated in two groups (*n* = 8/group) as follows: GK vehicle group and GK + EA group (50 mg/kg/day). EA doses were selected based on previous studies [22]. Importantly, EA has low bioavailability in water solution [23]. To rule out this issue, the EA solution was prepared in 10% methyl-beta-cyclodextrin to enhance its bioavailability [22]. EA was orally administrated daily by gavage for 28 days. The control GK rats received the same volume of vehicle (10% methyl-beta-cyclodextrin) by oral gavage throughout the entire experimental protocol. The no observed adverse effect level (NOAEL) of EA was assessed to be 3826 mg/kg body weight/day in female rats [24,25]; therefore, the dose selected for this study was safe. Body weights were monitored weekly throughout the treatment period. All experiments and protocols were conducted in accordance with the National Institute of Health (NIH) Guidelines for the Care and Use of Laboratory Animals and approved by the New York Institute of Technology College of Osteopathic Medicine (NYIT-COM) Animal Care and Use Committee.

### 2.3. Measurement of Plasma Metabolic Parameters and Tissue Collection

After 10 h fasting, blood glucose was measured before, during and after the treatment, through capillary blood drops obtained from the tail. Samples were analyzed with an AimStrip Plus glucometer from Germaine Labs (Indianapolis, IN, USA). At the time of the terminal experiments, rats were placed on a warm pad, under anesthesia (isoflurane 5% with flow of oxygen 1 L/min). After confirming that the rats were not responding to pain (toes pinch), an incision was made in the thoracic cavity where blood was collected directly from the heart. Then, the liver, retroperitoneal and periuterine fat pads were collected for weighing and storage (−80 °C). Blood samples were centrifuged at 1400 rpm for 15 min at room temperature. Supernatant, containing serum, was separated and used to determine levels of triglycerides (TG) and total cholesterol (TC) using commercial kits from Pointe Scientific (Canton, MI, USA), according to manufacturer’s instructions. Insulin levels were determined by using an ultra-sensitive rat insulin from Crystal Chem ELISA Kit (Downers Groce, IL, USA).

### 2.4. Glucose Tolerance and Insulin Sensitivity

Before and at the end of the experimental protocol with EA, rats were submitted to 10 h of fasting prior to an Oral Glucose Tolerance Test (oGTT). Rats received, by oral gavage, 2 g/kg body weight glucose solution. Blood drops were collected from the tail vein immediately before (time 0) and 15, 30, 60, and 120 min after the administration of glucose solution for blood glucose measurements, using an AimStrip Plus glucometer (Indianapolis, IN, USA). The Insulin Tolerance Test (ITT) measured insulin tolerance. The rats received an intraperitoneal injection of 1 IU/kg body weight of insulin (Novolin). Blood samples were obtained from the tail before (time 0) and 10, 30, and 45 min after insulin injection. The glucose disappearance rate (kITT) was calculated based on ITT data; it was calculated as 0.693/t_1/2_, where t_1/2_ is half the time taken to reach maximum blood glucose decay [26]. Insulin resistance was evaluated using the homeostasis model assessment (HOMA) index of insulin resistance (HOMA-IR = fasting insulin (µU/mL)/fasting glucose (mM)/22.5) [27,28]. This has been previously validated for use in rodent studies [29,30,31,32,33].

### 2.5. Histological Analysis of Liver

At the terminal experiment, livers from the experimental groups were collected and immediately embedded in Tissue-Tek medium, frozen in a mixture of dry ice and 2-methylpentane. Then, 10-μm-thick transversal sections were obtained and fixed in 10% formalin, following the stained protocols for hematoxylin and eosin (H&E) and Oil Red O (Sigma-Aldrich, St. Louis, MO, USA). The H&E staining was used to conduct a morphological analysis of the NAFLD activity score (NAS) in the liver, where two independent researchers performed a double-blind analysis of the degrees of steatosis, hepatocellular ballooning and lobular inflammation [34]. Hepatic fat accumulation was detected by Oil Red O staining. Age matched non-diabetic Wistar rats were used as the control group. The images were captured with a 40× objective and quantified through ImageJ software (NIH).

### 2.6. Oxidative Stress in the Liver

Oxidative stress was determined in frozen and unfixed liver tissue sections by measuring levels of superoxide anion utilizing the fluorescent probe Dihydroethidium (DHE), as previously described [18]. Briefly, cryosections of livers from both groups (GK and GK + EA) were incubated with 25 µM DHE in a light-protected humidified chamber at 37 °C for 30 min and washed with PBS. DHE fluorescence was captured using an Olympus DP73 fluorescence microscope fitted with a camera and quantified as previously described [18]. The fluorescence intensity was quantified in five arbitrarily selected fields, and the mean value for each section was calculated. The results are expressed as percentage of fluorescence in the GK+EA in comparison to the GK group.

### 2.7. Immunoblotting

Protein expression was detected by Western blotting. Total protein was extracted from the livers of both experimental groups. Fifty (50) µg of hepatic tissue was separated by standard sodium dodecyl sulfate polyacrylamide gel electrophoresis (SDS-PAGE), transferred to polyvinylidene difluoride (PVDF) membranes, and immunoblotted with the following antibodies: total Akt (#9272, Cell Signaling Technology, Danvers, MA, USA), phospho-Akt Ser 473 (#9271, Cell Signaling Technology, Danvers, MA, USA), NRF2 (sc-722, Santa Cruz Biotechnology, Santa Cruz, CA, USA), superoxide dismutase-2 (SOD2) (#137037, Abcam, Cambridge, MA, USA), p47phox (sc-17845, Santa Cruz Biotechnology, Santa Cruz, CA, USA), HIF-α (#3716, Cell Signaling Technology, Danvers, MA, USA). The corresponding IgG-HRP secondary antibodies were used to detect primary antibodies (Cell Signaling Technology, Danvers, MA, USA). Membranes were stripped and re-probed with an internal loading control, β-actin (# 4967, Cell Signaling Technology, Danvers, MA, USA) or GAPDH (#2118, Cell Signaling Technology, Danvers, MA, USA). Protein bands were detected using Lumigen Enhanced Chemiluminescence (ECL) Ultra (Lumigen Inc., Southfield, MI, USA).

### 2.8. Statistical Analysis

All data is expressed as means ± SEMs. Results were analyzed using one-way ANOVA followed by Bonferroni’s post hoc test. Student’s *t* tests were used when appropriate. *p* < 0.05 was considered statistically significant. The “*n*” in each experiment represents the different biological replicates.

## 3. Results

### 3.1. Effect of EA on Metabolic Parameters and Insulin Sensitivity

**Figure 1:**
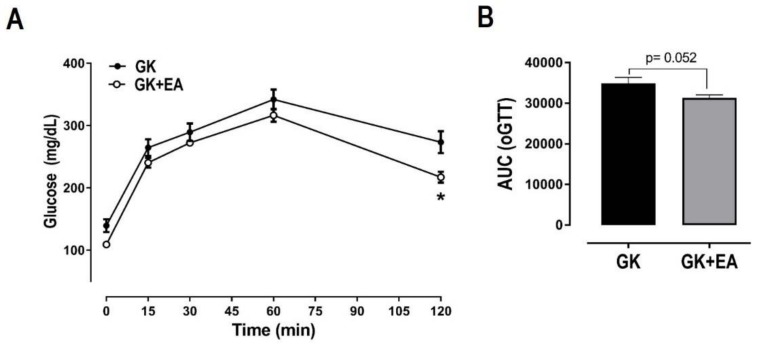
Effect of EA treatment on glucose tolerance in diabetic GK rats. (**A**) oGTT was performed in female diabetic GK rats with or without EA (50 mg/kg) treatment for 45 days. After 10 h fasting, rats received 2 mg/kg glucose solution by oral gavage and blood samples were collected at 15, 30, 45, 60, 75, 90, 105 and 120 min after the administration of glucose solution. Area under the curve of glucose measured during oGTT (**B**). Data are presented as mean ± SEM. (*n* = 6 per group); * *p* < 0.05 vs. GK without EA treatment.

**Figure 2:**
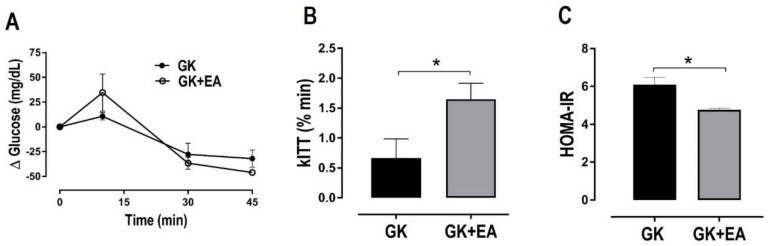
EA treatment enhances insulin sensitivity and reduces insulin resistance in diabetic GK rats. (**A**) Insulin Tolerance Test (**B**) Rate constant for the disappearance of glucose in insulin tolerance test (kITT) and (**C**) insulin resistance index estimation by homeostasis model assessment method (HOMA-IR) were performed in female diabetic GK rats with or without EA (50 mg/kg) treatment for 45 days. Data are presented as mean ± SEM, * *p* < 0.05, (*n* = 8).

EA did not alter body weight or periuterine fat, but it significantly reduced retroperitoneal fat in female diabetic GK rats (Table 1). No differences in lipid profiles, including triglycerides and total cholesterol, were found between the groups (Table 2). Fasting hyperglycemia and glucose intolerance are present in T2DM [35,36]. As expected, GK groups displayed increased levels of fasting blood glucose, which were significantly reduced with EA treatment (Table 2). Glucose tolerance was assessed by oGTT. The GK group maintained a high blood glucose concentration throughout the oGTT, characterizing impaired glucose tolerance (Figure 1). The GK rats treated with EA exhibited improved glucose tolerance in comparison to the GK group, only at the end of the experiment (120 min). EA markedly increased insulin sensitivity in GK rats, as evidenced by the kITT measurement (Figure 2A). To determine whether EA also affected insulin resistance, HOMA-IR was calculated. As shown in Figure 1B, EA significantly diminished insulin resistance in GK rats (Figure 2B).

### 3.2. EA Reduces Hepatic Steatosis and Oxidative Stress

**Figure 3:**
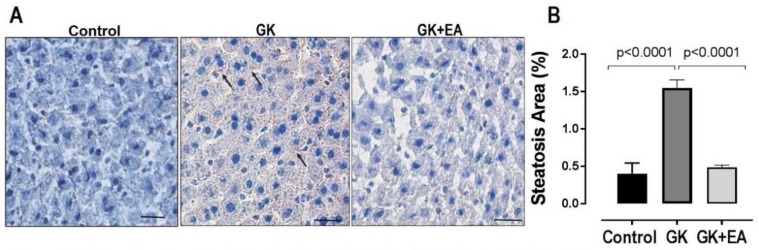
EA treatment reduces hepatic steatosis in diabetic GK Rats. Oil Red staining for detection of fat droplets was preformed in frozen sections of livers from age matched Wistar control, and diabetic GK rats with or without EA treatment (50 mg/kg) for 45 days. (**A**) Representative oil red staining showing steatosis (black arrows). Scale bar: 50 μm; (**B**) Morpho-quantitative analysis of hepatic steatosis area. Data are represented as mean ± SEM; * *p* < 0.05, (*n* = 6 per group).

**Figure 4:**
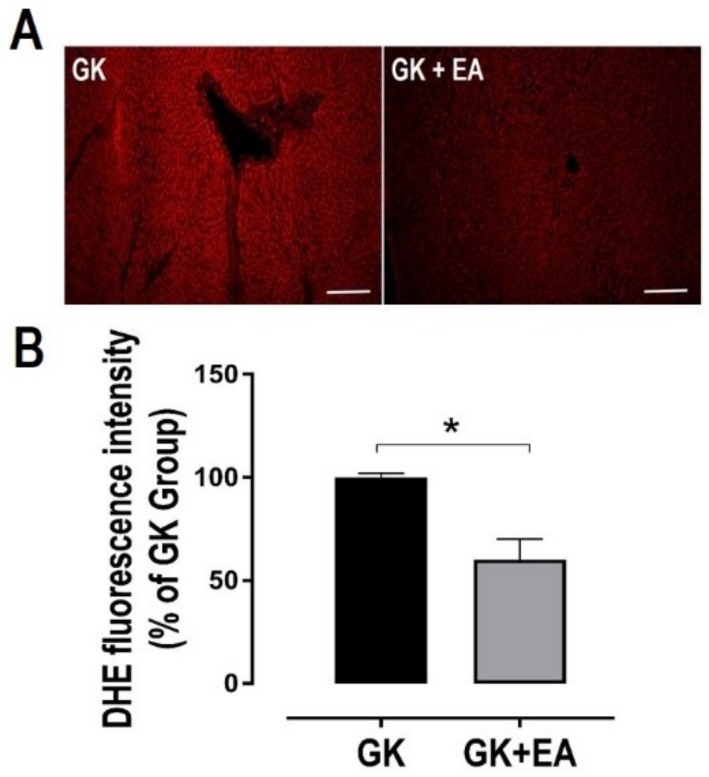
EA treatment reduces hepatic oxidative stress in diabetic GK rats. DHE staining was performed in frozen section of liver from diabetic GK rats with or without treatment with EA (50 mg/kg) for 45 days for detection of superoxide anion. (**A**) Representative image of DHE staining in liver section. Scale bar: 50 μm; (**B**) Quantitative analysis of DHE fluorescence. Data are presented as mean ± SEM; * *p* < 0.05, (*n* = 6 per group).

**Figure 5:**
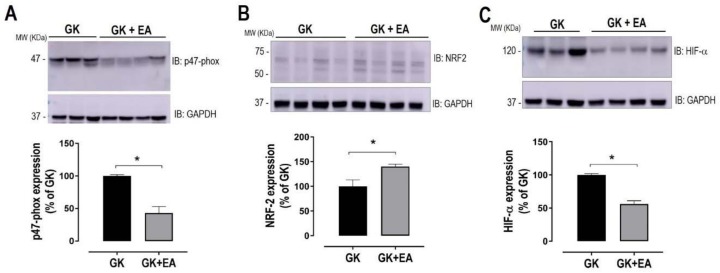
EA treatment reduces stress reduces stress machinery in diabetic livers. Expression of p47-phox (**A**), antioxidant transcription factors, NRF2 (**B**) and HIF-α (**C**) and in hepatic homogenate from diabetic GK rats with or without treatment with EA (50 mg/kg) for 45 days. Top panels: representative blots. Bottom panels: densitometry analysis. GAPDH was used as loading control. Data are presented as mean ± SEM; * *p* < 0.05, (*n* = 6 per group).

Dysregulation in the lipid metabolism in the livers of GK rats has been reported [37]. However, the accumulation of fat in the hepatic tissue of GK rats remains controversial. Therefore, fat accumulation in the livers of GK rats was evaluated using Oil Red O staining. As shown in Figure 3, diabetic GK rats at approximately 11 months of age exhibited increased fat droplet accumulation, which was significantly reduced with EA treatment. To determine whether hepatic steatosis is associated with an increase in oxidative stress in diabetic GK rats, the superoxide anion levels in liver sections from both experimental groups were evaluated. GK rats treated with EA exhibited a significant decrease in hepatic oxidative stress, as shown in Figure 4. Next, to determine how EA decreased oxidative stress in the livers of diabetic rats, the expressions of potential proteins associated with the oxidative pathway were quantified. EA markedly decreased the expression of NADPH oxidase p47phox (Figure 5A) and increased the expression of NRF-2, an antioxidant transcription factor, (Figure 5B) in livers from GK rats. Moreover, EA treatment reduced the expression of HIF-α (Figure 5C), an intrinsic marker for tissue hypoxia related to oxidative stress in the liver [38].

### 3.3. EA Ameliorates Hepatic Insulin Signaling

**Figure 6:**
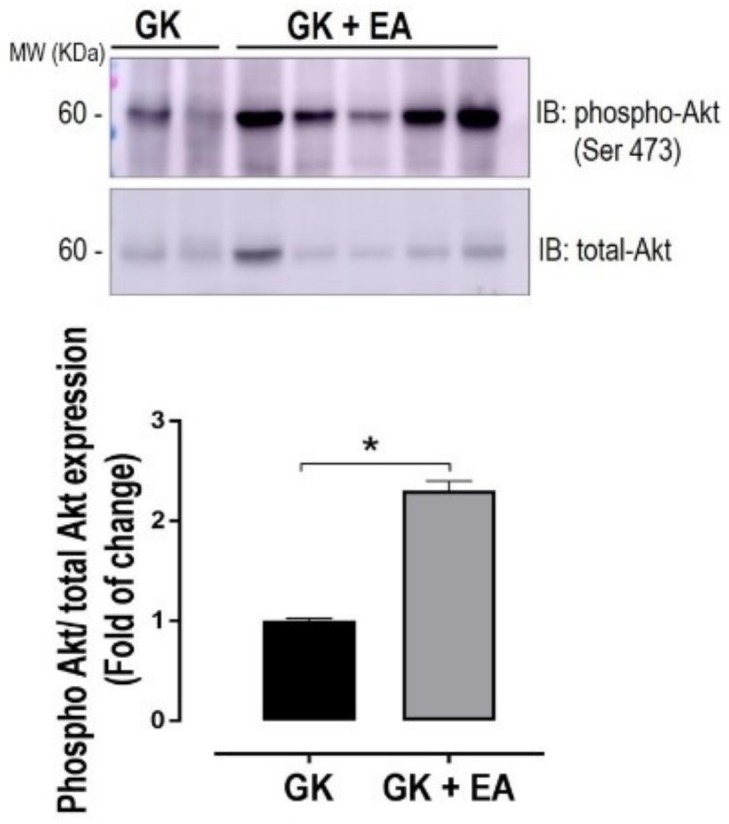
EA treatment ameliorates hepatic insulin signaling. Expression of phosphorylated Akt at serine 473 in hepatic homogenate from diabetic GK rats with or without treatment with EA (50 mg/kg) for 45 days. Top panel: representative blot. Bottom panel: densitometry analysis. Data are presented as mean ± SEM; * *p* < 0.05, (*n* = 6 per group).

Together with hepatic oxidative stress, hepatic insulin resistance plays a crucial role in the pathogenesis of NAFLD [39]. Moreover, the impairment of Akt signaling is closely linked with hepatic insulin resistance [40]. Therefore, in order to determine whether EA improves insulin signaling in diabetic livers, Akt activation was assessed. As shown in Figure 6, EA significantly activated Akt signaling in liver from GK rats, as demonstrated by increased levels of phosphorylated Akt.

## 4. Discussion

It is well recognized that NAFLD is a major complication in T2DM. Oxidative stress has been identified as a key mechanism driving NAFLD in T2DM, by inducing severe alterations in lipid metabolism [41]. Therefore, antioxidant treatments are an attractive therapeutic approach to target diabetic hepatic complications. Evidence has supported EA as a potent natural polyphenolic antioxidant with beneficial effects in the liver and cardiovascular system [42,43]. This is further supported by our recent studies which showed that EA reduces the vascular oxidative stress induced by high glucose [18]. Accordingly, the rationale for the present study was to determine whether EA also reduces oxidative stress in diabetic liver, thus improving NAFLD.

The main finding of the present study was that treatment with EA attenuated hepatic steatosis in association with reduced oxidative stress in diabetic GK rats. Mechanistically, EA decreased p47phox and increased antioxidant NRF2 expression, implying that EA exerts its antioxidant effect in the liver by targeting p47phox and NRF2. Our results are consistent with a recent study showing that EA diminishes oxidative stress in hypertensive animals via downregulation of p47phox in the vasculature [44]. Moreover, activation of NRF2, a major antioxidant transcription factor, has been reported to mediate the antioxidant effect of EA [45,46]. Several studies have demonstrated that EA increases NRF2 protein levels; however, the mechanisms by which this occurs remain unknown [46,47]. A limitation of our study is that we did not determine the mechanism by which EA increases NRF2 protein expression. As a transcriptional factor, NRF2 is regulated at several levels, including transcription, degradation, translocation and post-translational modification, such as phosphorylation [48,49,50]. The activity of NRF2 is primarily regulated via its interaction with Keap1 (Kelch-like ECH-associated protein 1), which directs the transcription factor to proteasomal degradation [51]. Additionally, growing evidence has also shown that NRF2 is not only involved in antioxidant defenses but also has a protective role against steatosis by repressing SREB-1c expression. This enhances fatty acid oxidation and antagonizes inflammation in hepatocytes [52,53].

NRF1, a sister molecule of NRF2, has been recently identified as a regulator of hepatic lipid metabolism and systemic insulin resistance [54,55,56,57]. It has been demonstrated that NRF1-deficient mice livers display decreased expression of genes related to lipid metabolism, suggesting that together with NRF2, NRF1 also plays an important role in hepatic lipid metabolism via its binding to antioxidant response elements (ARE) [55].

Increased ROS-induced oxidative stress activates HIF-α, a transcription factor implicated in hypoxia and hepatic lipid accumulation [14,15]. In fact, hypoxia has been demonstrated to play an important role in the development and progression of NAFLD as a result of increased oxygen consumption and metabolic demands [13]. In this study, we found that EA decreased HIF-α expression in the liver of diabetic GK rats, which likely can explain the effect of EA in reducing hepatic steatosis. The novelty of our study is that we demonstrated that the p47phox, NRF2 and HIF-α pathway is a potential mechanism by which EA exerts its antioxidant effects in diabetic livers.

The GK rat is a non-obese model of T2DM that has been widely utilized to investigate human diabetic complications. It exhibits elevated fasting blood glucose and an impaired response to glucose [58,59]. As expected, we found that GK rats exhibited high levels of fasting blood glucose as well as intolerance to glucose, which were significantly attenuated with EA treatment. These results confirm previous findings showing an anti-hyperglycemic effect of EA in diabetic animals [60]. The mechanisms by which EA exerts its anti-diabetic effects remain unknown. EA also possesses triglyceride-lowering properties [61]. However, we did not observe a difference in triglyceride levels in GK rats treated with EA compared to those not treated with EA. A possible explanation for these differences may be due to the fact that the previous study utilized a long-term treatment protocol with EA, while we used a short-term protocol. Future studies using a longer period of EA treatment will be needed.

A relationship between insulin resistance and NAFLD has been previously described and suggests that therapies targeting an increase in insulin sensitivity may be beneficial for the prevention of the development of NAFLD [62]. We found that EA improved insulin sensitivity and decreased insulin resistance as determined by kITT and HOMA-IR, respectively. Of note, several studies have now used HOMA-IR to assess rodent insulin sensitivity. Overall, HOMA-IR provides an acceptable measurement of insulin resistance when applied to rats and mice as it does in humans [29,33]. To assess whether this systemic improvement in insulin resistance caused by EA also extends to hepatic tissue, we evaluated the activation of Akt, a key molecule in the insulin signaling pathway [63]. Treatment with EA markedly increased phosphorylation of Akt in diabetic livers, indicating that EA ameliorates hepatic insulin resistance. However, it should be noted that our Akt measurements were conducted under basal non-stimulated conditions. Although impaired, Akt phosphorylation is detected under basal conditions and responds to increasing insulin levels at a lesser but parallel extent to that seen in Wistar control rats [64]. Therefore, since our study used only GK rats, which were either treated with Ellagic acid or not treated, increased Akt phosphorylation might certainly be ascribed to improved insulin sensitivity. This assumption is further supported by the fact that fasting serum insulin levels did not differ between the groups (Table 2). Moreover, measurements of Akt phosphorylation at basal non-stimulated conditions have been reported by others [65,66,67,68]. Collectively, our data demonstrates that EA exerts anti-diabetic effects, consistent with previous findings [21,60,69,70].

An unexpected finding of our study was that EA reduced the amount of retroperitoneal fat in diabetic GK rats. A prior study has shown that treatment with a freeze-dried strawberry-blueberry mixture significantly reduced adipogenesis and lipogenesis mediators, leading to decreases in white adipose tissue depots, such as retroperitoneal fat [71]. Given that EA has been shown to be present in berries, we speculate that reductions in lipogenesis and adipogenesis may be potential mechanisms by which treatment with EA reduces retroperitoneal fat in GK rats. Of note, a recent study has provided evidence that EA plays an important role in adipose tissue metabolism, conferring its lipid-lowering effects [72].

It is unlikely that diabetic GK rats, as a non-obese model, would have extensive fat accumulation in the liver. Data on fat accumulation in the hepatocytes of GK rats is controversial since the majority of the studies utilize young GK rats, which, for the most part, do not exhibit diabetic complications. Not surprisingly, the majority of published studies in GK rats have not reported a significant presence of hepatic lipid accumulation on these animals [32,37,73]. In contrast to prior studies, we found significant fat accumulation in the livers of diabetic GK rats. Therefore, the main reason underlying this discrepancy may the advanced age of the GK rats that we utilized in this study. Since we aimed to study long-term hepatic complications caused by T2DM, we selected older GK rats that more closely mimic an adult individual with T2DM. Furthermore, studies have demonstrated that chronic hyperglycemia leads to an accumulation of fat within hepatocytes, which can progress to non-alcoholic steatohepatitis, advanced fibrosis and cirrhosis, as well as a number of other extra hepatic complications. These studies have described a tight relationship between hyperglycemia and NAFLD prevalence [74,75]. Given that the GK rats utilized in this study exhibited chronic hyperglycemia associated with hepatic oxidative stress, it is more likely that fat accumulation in the livers of these animals would occur, as we observed.

Using acute incubation of blood vessels with EA, we recently reported that EA improves high glucose-induced vascular dysfunction by diminishing oxidative stress [18]. To further confirm our previous findings and address the hypothesis that treating NAFLD in T2DM would consequently improve vascular outcomes, we evaluated arterial blood pressure and vascular reactivity in diabetic GK rats treated with EA. Surprisingly, the increased blood pressure and impaired vasodilatation that was present in diabetic GK rats remained unchanged with EA treatment, contrary to our hypothesis that the improvement of NAFLD would result in better vascular outcomes in diabetic rats (data not shown). A possible explanation for this lack of vascular effect is that these diabetic GK rats were treated with EA in a short-term protocol. Although the time frame of treatment with EA was sufficient to improve hepatic outcomes, it is likely that the same time frame does not apply for vascular dysfunction. Future studies are needed to evaluate the effects of a long-term treatment with EA on vascular dysfunction.

In summary, our results reveal a novel effect of EA, whereby it reduces hepatic steatosis and oxidative stress, and improves insulin sensitivity. Specifically, we found that EA exerts its antioxidant effects via the downregulation of p47phox and upregulation of NRF2 in livers from diabetic rats, which ultimately decreases HIF-α expression. Further, EA decreased levels of blood glucose, and improved systemic and hepatic insulin resistance, as evidenced by the increased phosphorylation of Akt. Our data provides novel insights into the benefits of oral EA for the treatment of hepatic complications in T2DM. Dietary supplementation of this substance has the potential to be used as a supplemental therapy alongside current diabetic treatment plans, with the goal of reducing the progression of liver disease in diabetic patients. This data supports further investigation of this compound given its potential for use in the clinical setting.

## Figures and Tables

**Table 1:** Morphometric parameters of diabetic female Goto Kakizaki (GK) rats after oral treatment with Ellagic Acid (EA) (50 mg/kg) for 45 days. (*n* = 6 per group)

Parameter	GK	GK+EA
Body Weight, BW (g)	257.50 ± 10.35	256.50 ± 11.59
Liver (g/100 g BW)	2.49 ± 0.04	2.54 ± 0.07
Kidney (g/100 g BW)	0.67 ± 0.02	0.69 ± 0.01
Retroperitoneal Fat (g/100 g BW)	1.65 ± 0.14	1.14 ± 0.12 *
Periuterine (g/100 g BW)	1.20 ± 0.26	0.76 ± 0.06

* *p* < 0.05 vs. GK (Student’s *t* test).

**Table 2:** Glucose and lipid parameters of diabetic female GK rats after oral treatment with Ellagic Acid (EA) (50 mg/kg) for 45 days. (*n* = 6 per group)

Parameter	GK	GK+EA
Fasting Blood Glucose (mg/dL)	139.30 ± 10.35	109.00 ± 3.60 *
Insulin (ng/mL)	0.69 ± 0.06	0.61 ± 0.01
Total Cholesterol (mg/dL)	49.30 ± 6.42	48.10 ± 3.29
Triglycerides (mg/dL)	86.11 ± 8.62	83.61 ± 10.36

* *p* < 0.05 vs. GK (Student’s *t* test).

## Abbreviations

NAFLD	Non-Alcoholic Fatty Liver Disease
T2DM	Type 2 Diabetes Mellitus
HOMA IR	Homeostasis Model Assessment Index of Insulin Resistance
H&E	Hematoxylin and eosin
NRF2	NF-E2-related factor-2
EA	Ellagic acid
GK	Goto Kakizaki
HIF-α	Hypoxia-inducible factor-α
ROS	Reactive oxygen species
TG	Triglycerides
TC	Total cholesterol
oGTT	Oral Glucose Tolerance Test
ITT	Insulin Tolerance Test
kITT	Glucose disappearance rate
HOMA	Homeostasis model assessment
NAS	NAFLD activity score
DHE	Dihydroethidium
SBP	Systolic blood pressure
Ach	Acetylcholine
p47phox	NADPH oxidase p47phox
SOD2	Superoxide dismutase-2
NOAEL	No Observed Adverse Effect Level
PE	Phenylephrine
